# Using tears as a non-invasive source for early detection of breast cancer

**DOI:** 10.1371/journal.pone.0267676

**Published:** 2022-04-26

**Authors:** Anna Daily, Prashanth Ravishankar, Steve Harms, V. Suzanne Klimberg

**Affiliations:** 1 Namida Lab Inc, Fayetteville, Arkansas, United States of America; 2 The Breast Center-Medical Associates of Northwest Arkansas, Fayetteville, Arkansas, United States of America; 3 Department of Surgery, University of Texas Medical Branch, Galveston, Texas, United States of America; 4 Breast Surgical Oncology, The University of Texas MD Anderson Cancer Center, Houston, Texas, United States of America; CNR, ITALY

## Abstract

The changing expression levels of ocular proteins in response to systemic disease has been well established in literature. In this study, we examined the ocular proteome to identify protein biomarkers with altered expression levels in women diagnosed with breast cancer. Tear samples were collected from 273 participants using Schirmer strip collection methods. Following protein elution, proteome wide trypsin digestion with Liquid chromatography/tandem mass spectrometry (LC-MS/MS) was used to identify potential protein biomarkers with altered expression levels in breast cancer patients. Selected biomarkers were further validated by enzyme linked immunosorbent assay (ELISA). A total of 102 individual tear samples (51 breast cancer, 51 control) were analyzed by LC-MS/MS which identified 301 proteins. Spectral intensities between the groups were compared and 14 significant proteins (p-value <0.05) were identified as potential biomarkers in breast cancer patients. Three biomarkers, S100A8 (p-value = 0.0069, 7.8-fold increase), S100A9 (p-value = 0.0048, 10.2-fold increase), and Galectin-3 binding protein (p-value = 0.01, 3.0-fold increase) with an increased expression in breast cancer patients were selected for validation using ELISA. Validation by ELISA was conducted using 171 individual tear samples (75 Breast Cancer and 96 Control). Similar to the observed LC-MS/MS results, S100A8 (p-value <0.0001) and S100A9 (p-value <0.0001) showed significantly higher expression in breast cancer patients. However, galectin-3 binding protein had increased expression in the control group. Our results provide further support for using tear proteins to detect non-ocular systemic diseases such as breast cancer. Our work provides crucial details to support the continued evaluation of tear samples in the screening and diagnosis of breast cancer and paves the way for future evaluation of the tear proteome for screening and diagnosis of systemic diseases.

## Introduction

With advances in screening techniques, and adjustment of recommended screening guidelines, mortality rates due to breast cancer continue to drop. Despite the estimated drop-in mortality rates, breast cancer still remains the highest cancer diagnosis of women globally [1]. While the United States spends more on cancer screening than any other industrialized country, we also have the lowest life expectancy [2]. While family history remains one of the most significant risk factors, the list of factors classifying an individual as “high-risk” continues to grow. Interestingly, family history of breast cancer increases a woman’s chance of developing breast cancer by almost two-fold, however less than fifteen percent of all breast cancer diagnosis are attributed to women with family history [3–5].

As research continues to unfold, additional risk factors such as birth control use, hormone replacement therapy, breast tissue density, and obesity continue to increase the number of women who are classified as high-risk [6]. Massive efforts are currently focused on developing a personalized risk-based screening approach that considers individual biological characteristics, circumstances, and lifestyles [7,8]. Results from these studies could allow justification of focusing the most intensive screening on the portion of the population at the highest known risk of cancer formation. Biological tests could play an important role in future cancer screening risk stratification.

With continued advancement in biomarker identification techniques, there is increasing interest in finding markers of disease in non-traditional biological fluids. Breast cancer associated biomarkers have been identified in urine [9], nipple fluid aspirate [10], as well as breast milk [11]. The knowledge gained from identification of disease markers in fluids other than those traditionally associated with cancer diagnosis could improve the ability to not only understand disease instigation and progression, but also narrow the field of who truly needs to be considered high risk.

Biological fluids such as blood and urine have been extensively studied for their clinical value; however, tear fluid is one of the most underrated biofluids that has been gaining interest in recent years [12]. In this study, tears were used as a source for non-traditional biological fluid that could expand upon our current knowledge on crucial breast cancer biomarkers. Tears are transparent, extracellular fluid secreted by the lacrimal glands forming a mechanical and antimicrobial layer protecting the ocular surface [13]. They are comprised mainly of water and electrolytes but also contains a vast range/multitude of/hundreds of proteins/peptides, lipids, glycoproteins, hormones, and small molecule metabolites [12,13]. The importance of tear analysis extends beyond the ocular surface as they are secreted by the lacrimal glands in the eyelids through filtration from blood plasma and can provide valuable/relevant clinical information from unrelated body parts [14,15]. Studies have focused on using tears as a non-invasive source to conduct biomarker discovery studies as a novel and reliable means to predict and diagnose diseases while also serving to monitor disease progression and therapy [16–25]. The simplicity of tear fluid collection and evaluation could potentially provide a convenient, non-invasive method of testing, fitting easily into a personalized risk-based medicine approach [12,20,26,27].

Ease of collection, high protein concentration, and lower complexity of the tear fluid compared to blood make tears an ideal diagnostic fluid [27–29]. Additionally, low molecular weight proteins are easily accessible in tear fluids and can aid in identifying crucial cancer biomarkers [26]. Several preliminary studies utilizing tear fluid have been conducted looking at systemic diseases without ocular diseases, such as cancer (breast, prostate, lung, ovary, and colon) [23,30–32] and neurological diseases (multiple sclerosis, Parkinson’s disease) [33–37]. Here data is presented to support using tear proteins to detect breast cancer. In this study, data collected from 273 individual utilizing the Schirmer strip method will be reported.

## Materials and methods

### Selection criteria and sampling methods

All protocols involving human subjects were reviewed and approved by the University of Arkansas IRB committee (13-11-289) prior to sample collection. The sampling technique used was a purposive, non-random sampling strategy to recruit women with the requisite inclusion criteria (Table 1). Tear fluid samples were collected from study participants recruited at five breast health and surgery clinics; The Breast Center, Fayetteville, AR, USA; Breast Surgery of Tulsa, Tulsa OK, USA; Knoxville Comprehensive Breast Center, Knoxville, TN, USA; PeaceHealth Southwest, Vancouver, WA, USA; and PeaceHealth St. John Medical Center, Longview, WA, USA. Written informed consent was obtained from all participants prior to sample collection. Participants were recruited from individuals having a yearly screening mammogram, individuals having a biopsy, and individuals recently diagnosed with breast cancer being evaluated for pre-surgical MRI evaluation. Once imaging results were obtained, samples were then classified as: control (normal imaging no biopsy) or diagnosed breast cancer pre-treatment (diagnosed by biopsy).

**Table 1:** Inclusion/Exclusion criteria.

**Breast Cancer**		
***Exclusion***	***Inclusion Pre-Biopsy Patient***	***Inclusion Breast Cancer***
<18 years of age OR >100 years of age	18–100 years of age	18–100 years of age
Concurrent eye infection or trauma	Presenting for the evaluation of an abnormal exam or test (mammogram, ultrasound, MRI, PET, etc.)- they may or may not have a mass present.	Have been diagnosed but have not received treatment.
Acute conjunctivitis	Presenting for the evaluation of a palpable lump or mass	
	Presenting with a mass may be pre- or post-biopsy as long as there is a portion of the mass remaining.	
**Control samples**		
***Exclusion***	***Inclusion***	
<18 years of age OR >100 years of age	18–100 years of age	
Concurrent eye infection or trauma	Do not currently have or are being treated for breast cancer.	
Acute conjunctivitis		

### Tear sample collection

**Fig 1:**
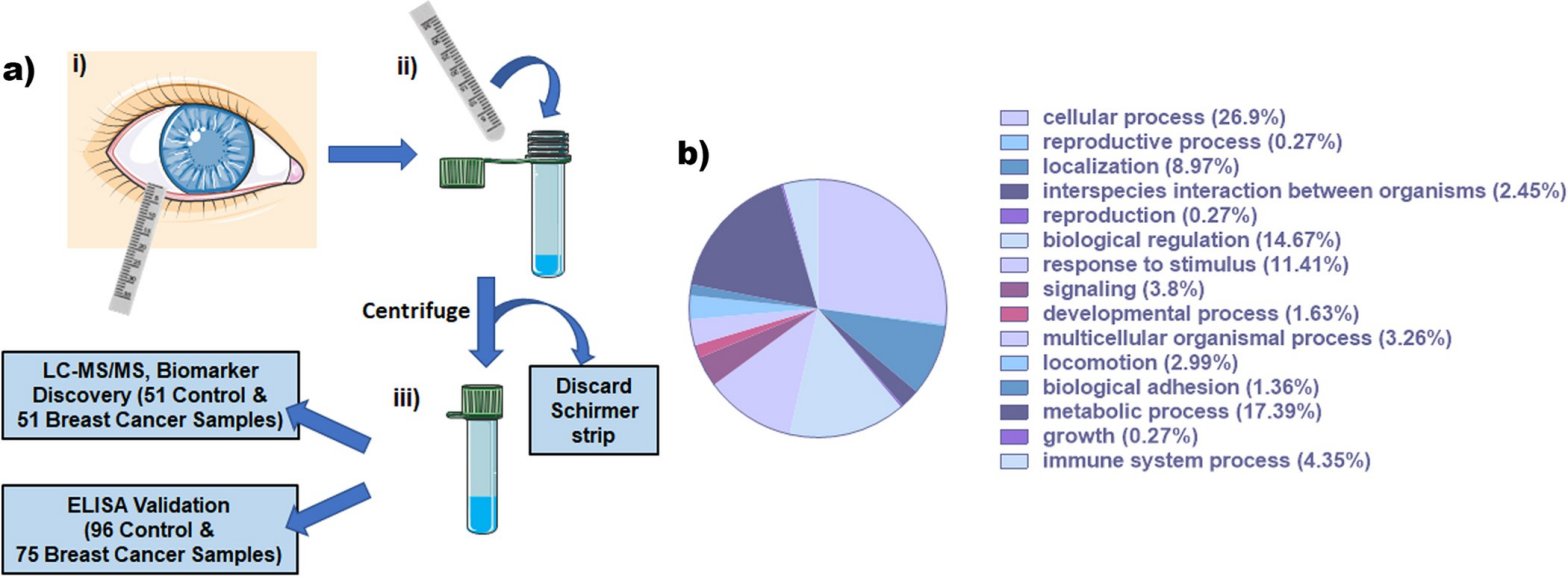
a) Schematic of Tear Collection using Schirmer strip- i) Schirmer strip is placed in the lower conjunctival fornix; ii) wetted strips are placed in screw-top tube prefilled with 225μL of 1XPBS and centrifuged to collect the tears; iii) Schirmer Strips are discarded to collect tears and stored in -80°C before being analyzed by LC-MS/MS and validated by ELISA. b) Functional classification of 301 mapped proteins in tear samples using PANTHER classification system.

Tear fluid samples were collected using Schirmer strips (Schirmer tear flow test strips, Eye Care and Cure Corp, Tucson, AZ, USA) from the lower conjunctival fornix. Once the Schirmer strip was in place (Fig 1A), the study participant was instructed to close their eyes and keep them closed until the fluid level reached the 25 mm mark or up to five minutes. Following sample collection, the strips were transferred into a 1.5 mL screw top tube containing 1X Phosphate Buffered Saline (1XPBS). Individual samples were centrifuged for 30 seconds using a super-spin mini centrifuge, the buffer was aliquoted and stored at -80°C until use.

### LC-MS/MS; Biomarker discovery

Total protein content was determined for each tear sample using bicinchoninic acid assay (BCA) (Thermo Scientific, Waltham, MA, USA) and samples containing 7μg of protein were prepped for in-solution digests. Solution digests were carried out on all tear fluid samples in 100 mM ammonium bicarbonate (Sigma-Aldrich, St. Louis, MO, USA), following reduction in 10 mM Tris[2-carboxyethyl]phosphine (Pierce, Waltham, MA, USA) and alkylation in 50 mM iodoacetamide (Sigma-Aldrich) by addition of 100 ng porcine trypsin (Promega, Madison, WI, USA) and incubation at 37°C for 12–16 hours. Peptide products were then acidified in 0.1% formic acid (Fluka, Honeywell Research Chemicals, Morris Plains, NJ, USA). Tryptic peptides were separated by reverse-phase Jupiter Proteo resin (Phenomenex, Torrance, CA, USA) on a 100 x 0.075 mm column using a nanoAcquity UPLC system (Waters Corporation, Milford, MA, USA). Peptides were eluted using an 80 min gradient from 97:3 to 35:65 buffer A:B ratio [Buffer A = 0.1% formic acid, 0.05% acetonitrile; buffer B = 0.1% formic acid, 75% acetonitrile]. Eluted peptides were ionized by electrospray (1.8 kV) followed by MS/MS analysis using collision-induced dissociation on an LTQ Orbitrap Velos mass spectrometer (Thermo Scientific, Waltham, MA, USA). MS data was acquired using the FTMS analyzer in profile mode at a resolution of 60,000 over a range of 375 to 1500 m/z. MS/MS data was acquired for the top 15 peaks from each MS scan using the ion trap analyzer in centroid mode and normal mass range with a normalized collision energy of 35.0. Proteins were identified from MS/MS spectra using the Mascot search engine (Matrix Science, Boston, MA, USA) or MaxQuant quantitative proteomics software (Max Planck Institute, Munich, Germany) and the search results were compiled using Scaffold (Proteome Software, Portland, OR, USA). The protein and peptide threshold filters were set at 99% and 95% respectively, with a minimum peptide number of 2.

### ELISA biomarker validation

Standard sandwich ELISA procedures using DuoSets ELISA kits purchased from R&D Systems (Minneapolis, MN, USA) were used to evaluate the expression level of S100A8 (SA8), S100A9 (SA9), and Galectin-3-Binding Protein (LG3BP) in tear samples. Assays were conducted according to the manufacturer’s guidelines. Based on results from previous optimization tests, tear samples were diluted at 1:10 for SA8 and SA9 analytes and 1:50 for LG3BP. All samples and standards were tested in duplicate. The absorbance was read at 450 nm and 570 nm using a Synergy LX microplate reader (BioTek, Winooski, VT, USA). Absorbance at 570 nm was subtracted from 450 nm for each well. ELISA data was analyzed using Prism version 6.0 (GraphPad, San Diego, CA, USA).

### Statistical analysis

For protein discovery, variations in spectral intensities of tryptic fragments mapped to protein IDs in Scaffold software were compared by utilizing One-Way ANOVA in JMP Pro11 software package. Predicted protein intensities were assessed across groups to elucidate potential biomarkers. An alpha level of 0.05 was used as an indicator of significant expression change between the groups. The functional categories of the identified proteins were determined using an online protein annotation tool, PANTHER (protein annotation through evolutionary relationship, www.pantherdb.org). R statistical software was used to apply logistic regression to protein concentrations determined by ELISA for breast cancer versus control. The probability of breast cancer as a function of protein concentration was obtained and used to create a decision rule. The decision rule was then used to classify each case via the confusion matrix and related metrics such as sensitivity, specificity, receiver operator characteristics (ROC), and area under the curve (AUC).

## Results

### Sample characteristics

Patient demographics for samples used in LC-MS/MS and ELISA are presented in Table 2. All data sets combined consisted of 273 participants- 102 samples were used for LC-MS/MS analysis (51 breast cancer, 51 control) and 171 for ELISA (75 Breast Cancer and 96 Control). Participants ranged from 23–91 years of age with an average age of 53.75 ± 14.2 years. Most participants were Caucasian (84.56%), followed by African American (3.52%) and Hispanic (1.86%). A modest percentage of participants had a family history of breast cancer (30.31%), and a small portion had a previous history of breast cancer (7.02%). Individuals with a previous history of breast cancer were considered acceptable for the control group if they were no longer undergoing treatment and had not undergone treatment for at least five years and had been returned to standard yearly screening. Of those study subjects for whom breast density was obtained, 57.5% had dense breast tissue. The abbreviation NR was used when clinical or demographic data was not reported by the participating clinical partner.

**Table 2:** Population demographics of tear samples used for LC-MS/MS and ELISA.

		LC-MS/MS				ELISA		
		No. of Patients, %				No. of Patients, %		
		Breast Cancer (N = 51)	Control (N = 51)	Total (N = 102)	Breast Cancer (N = 75)		Control (N = 96)	Total (N = 171)
**Age, y**								
	<39	2 (3.9)	12 (23.53)	14 (13.73)		2 (2.67)	15 (15.62)	17 (9.94)
	40–49	10 (19.6)	6 (11.76)	16 (15.67)		12 (16)	16 (16.67)	28 (16.38)
	50–59	12 (23.5)	14 (27.45)	26 (25.50)		23 (30.67)	26 (27.08)	49 (28.65)
	60–69	16 (31.4)	6 (11.76)	22 (21.57)		16 (21.33)	13 (13.54)	29 (16.97)
	>70	11 (21.6)	3 (5.89)	14 (13.73)		13 (17.33)	4 (4.17)	17 (9.94)
	NR	-	10 (19.61)	10 (9.80)		9 (12)	22 (22.92)	31 (18.13)
**Race**								
	African-American	2 (3.92)	2 (3.85)	4 (3.93)		2 (2.67)	4 (4.17)	6 (3.51)
	Asian	1 (1.96)	0 (0.0)	1 (0.98)		1 (1.33)	0 (0.0)	1 (0.58)
	Caucasian	46 (90.2)	43 (82.69)	89 (87.25)		63 (84)	74 (77.08)	137 (80.12)
	Hispanic	1 (1.96)	1 (1.92)	2 (1.96)		1 (1.33)	2 (2.08)	3 (1.75)
	Native Hawaiian or PI	0 (0.0)	0 (0.0)	0 (0.0)		2 (2.67)	0 (0.0)	2 (1.17)
	NR	1 (1.96)	5 (9.62)	6 (5.88)		6 (8)	16 (16.67)	22 (12.87)
**History of Breast Cancer**								
	Yes	7 (13.72)	5 (9.80)	12 (11.76)		5 (6.67)	0 (0.0)	5 (2.29)
	No	41 (80.39)	26 (50.98)	67 (65.69)		70 (93.33)	96 (100)	166 (97.08)
	NR	3 (5.89)	20 (39.22)	23 (22.55)		-	-	-
**Family History of Breast Cancer**								
	Yes	22 (43.14)	10 (19.61)	32 (31.37)		29 (38.67)	21 (21.87)	50 (29.24)
	No	29 (56.86)	20 (39.22)	49 (48.04)		42 (56)	48 (50)	90 (52.63)
	NR	0 (0.0)	21 (41.17)	21 (41.17)		4 (5.33)	27 (28.13)	31 (18.13)
**Breast Density**		(N = 30)	(N = 22)	(N = 52)		(N = 49)	(N = 64)	(N = 113)
	Fatty	1 (3.33)	2 (9.10)	3 (5.77)		1 (2.04)	5 (7.81)	6 (5.31)
	Scattered fibroglandular densities	13 (43.33)	15 (68.18)	28 (53.84)		21 (42.86)	35 (54.69)	56 (49.56)
	Heterogeneously Dense	14 (46.67)	4 (18.18)	18 (34.62)		24 (48.97)	20 (31.25)	44 (38.94)
	Extremely Dense	2 (6.67)	1 (4.54)	3 (5.77)		3 (6.12)	4 (6.25)	7 (6.19)

NR—No clinical or demographic data were reported.

Study enrollment was not limited to a particular type of breast cancer. This breast cancer sample group included IDC (57.14%), ILC (7.14%), DCIS (24.60%), as well as Metaplastic and Mucinous Carcinoma (1.59%) (Table 3). Grade designation was received for 90 of the 126 samples (Grade I 15.56%, Grade II 54.44%, Grade III 46.83%) (Table 3).

**Table 3:** Distribution of breast cancer types and grade designations.

LC-MS/MS Sample Pool			ELISA Sample Pool		
		No. of Patients (%)			No. of Patients (%)
**Cancer type**			**Cancer type**		
	IDC	28 (54.91)	** **	IDC	44 (58.67)
	ILC	4 (7.84)	** **	ILC	5 (6.67)
	DCIS	13 (25.49)	** **	DCIS	18 (24)
	IDC/DCIS	3 (5.88)	** **	IDC/DCIS	4 (5.33)
	Other	1 (1.96)	** **	Other	1 (1.33)
	NR	2 (3.92)	** **	NR	3 (4)
**Grade**			**Grade**		
	I	7 (13.73)	** **	I	7 (9.33)
	II	19 (37.25)		II	30 (40)
	III	13 (25.49)		III	20 (26.67)
	NR	12 (23.53)		NR	18 (24)

NR—No clinical or demographic data were reported.

### LC-MS/MS

In-solution trypsin digestion followed by LC-MS/MS was conducted and tryptic fragments were mapped for 301 proteins. The functional classifications of these identified proteins (Fig 1B) were primarily involved in cellular (26.9%) and metabolic (17.39%) processes, as well as biological regulation (14.67%). Spectral intensities were imported into JMP Pro11 software for One-way ANOVA and linear regression analysis. An alpha level of 0.05 was used as an indicator of significant expression change between the groups as well as a fold change greater than 2. Variations in spectral intensities of tryptic fragments were evaluated between control vs. breast cancer. Fourteen proteins (Table 4) were identified as potential biomarkers based on their significant p-values (p < 0.05) and fold changes; ACTN4, ADH1G, AK1C1, AL1A1, B4E1Z4, CYTN, G3P, K1C9, LDHA, LDHB, LG3BP, S100A8, S100A9, SPRL1. Of the fourteen proteins of interest, three proteins (S100A8, S100A9, and Galectin-3-binding protein) were selected as candidates for initial evaluation by ELISA based on observed fold change, statistical significance, and biological relevance. S100A8 and S100A9 had significantly higher expression levels with p-values of 0.0069 and 0.0048 in breast cancer patients, respectively, with an increased fold-change of 7.8 and 10.2 compared to controls. Similarly, Galectin-3-binding protein (LG3BP) had a 3-fold increase in expression (p-value = 0.01) compared to the control group.

**Table 4:** Summary of relevant biomarkers candidates from mass spec analysis.

Protein Name	Gene Name	Function	Cancer vs Control	Fold Change (elevated)
Alpha-actinin-4	ACTN4	Cell adhesion, cell migration, apoptosis regulation.	0.0443	2.3 (CRL)
Alcohol dehydrogenase 1C	ADH1G	Catalytic activity- ethanol, retinol, and other aliphatic alcohol metabolism.	0.0424	3.8 (CRL)
Aldo-keto reductase family 1 member C	AK1C1	Steroid hormone homeostasis, prostaglandin metabolism, metabolic activation of polycyclic aromatic hydrocarbons.	0.0256	3.17 (CRL)
Retinal dehydrogenase 1	AL1A1	Retinol metabolism, ethanol oxidation.	0.0325	1.77 (CRL)
Uncharacterized Protein	B4E1Z4		0.0334	1.7 (BC)
Cystatin-N	CYTN	Regulation of cysteine proteinases, antimicrobial, antiviral.	0.0355	1.68 (CRL)
Glyceraldehyde-3-phosphate dehydrogenase	G3P	Glycolysis, immune response, cytoskeleton organization, apoptosis.	0.0405	1.9 (CRL)
Keratin type 1 cytoskeletal 9	K1C9	Epidermis development, cytoskeletal structure integrity, keratin filament assembly.	0.0428	5.5 (BC)
L-lactate dehydrogenase A chain	LDHA	Oxidoreductase; Involved in the lactate and NAD metabolic process, positive regulation of apoptotic process.	0.0194	2.3 (CRL)
L-lactate dehydrogenase B chain	LDHB		0.0265	3.4 (CRL)
Galectin-3-binding protein	LG3BP	Immune system regulator, cell adhesion.	0.01	3.0 (BC)
S100 A8	S10A8	Inflammation, immune response, inhibitor of casein kinase.	0.0069	7.8 (BC)
S100 A9	S10A9		0.0048	10.2 (BC)
SPARC-like protein 1	SPRL1	Regulates ECM remodeling and cell-matrix interactions and angiogenesis.	0.0371	10.3 (BC)

### ELISA

**Fig 2:**
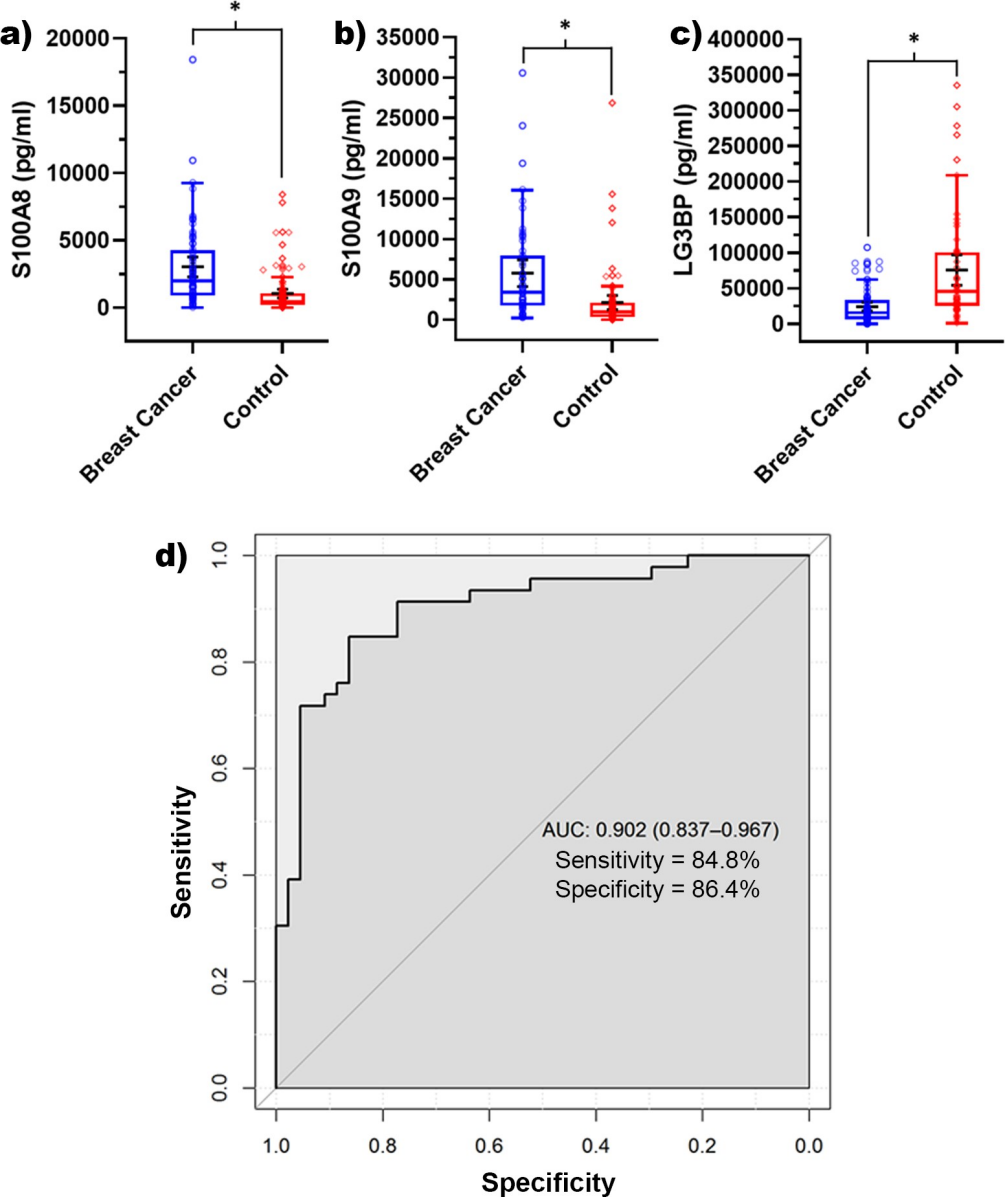
Investigation of biomarkers by ELISA- a) S100A8, b) S100A9, and c) LG3BP expression levels in tear samples between healthy and breast cancer women. (n = 96 control and 75 breast cancer samples, * indicates p < 0.0001); d) Receiver Operating Characteristics (ROC) curve for protein expression of potential breast cancer biomarkers. The area under the ROC curve (AUC) represents the accuracy of the combined potential biomarkers for distinguishing between the control and breast cancer sample groups.

Protein expression levels of S100A8, S100A9, and Galectin-3-binding protein were evaluated by ELISA. Concentrations of S100A8 and S100A9 were found to be elevated in breast cancer (mean concentration of 2997.17 pg/ml for S100A8 and 5729.19 pg/ml for S100A9) compared with the control group (mean concentration of 1003.92 pg/ml for S100A8 and 2107.35 pg/ml S100A9). Student t-test produced a p-value of <0.0001 indicating a statistically significant difference (Fig 2A and 2B). Galectin-3-binding protein was found to be increased in the control group, with a mean of 75263.2 pg/ml, compared to breast cancer group which had a mean of 23747.3 pg/ml and a p-value of <0.0001 (Fig 2C). The receiver operating characteristic (ROC) curve was generated (Fig 2D) using a linear logistic regression analysis, with an area under the curve (AUC) value of 0.902, a sensitivity of 84.8%, a specificity of 86.4% and an accuracy of 85.6%.

## Discussion

Here we demonstrate the utility of protein biomarkers isolated from tear samples to differentiate between individuals with a diagnosed breast cancer, a systemic non-ocular disease, and healthy individuals. A Schirmer strip was used as the sample collection source for tears as they can capture a large quantity of intracellular and extracellular proteins on the ocular surface compared to the other commonly used capillary tube method [38]. Total protein content of tear samples collected as determined by BCA, varied from 0.137–1.4 mg/ml. Overall, each sample had a more than adequate concentration of protein to be analyzed by LC-MS/MS. The decision was made to evaluate each sample independently rather than pooling samples to obtain a more accurate representation of the population. As stated earlier in the methods section, 7μg of proteins from each sample were analyzed using LC-MS/MS and tear proteins were identified using LC-MS/MS and mass intensities of associated peptide fragments were compared between the two groups to identify potential biomarkers. After evaluation, three proteins (S100A8, S100A9, and LG3BP) were selected for validation by performing ELISA based on previously reported association with breast cancer. Conducting validation utilizing a biological assay provided verification of the protein identification from LC-MS/MS.

The S100s are a family of Ca2+ binding proteins, with high sequence and folding similarity, involved in a wide range of biological processes such as proliferation, migration and/or invasion, inflammation and differentiation [39]. These proteins differ in shape and charge which contributes to a wide diversity of protein targets as well as a broad range of functions [40]. Elevated levels of S100A8 and S100A9 detected in tear samples from breast cancer patients supports previously reported results indicating elevated levels in serum and tissue of breast cancer patients [41–44]. A 2018 study reported an increased level of S100A8 expression levels in breast cancer patients with relapse and had significantly lower disease-free survival and overall survival durations [45]. The study further reported S100A8’s elevated levels in correlation with estrogen receptor-negative and triple-negative breast cancer clinical subtypes. S100A8 and S100A9 specifically have been shown to have altered expression levels in breast cancer tissues compared with normal tissues, with increased expression levels associated with non-functional BRCA1 (BReast CAncer gene 1) [40,46]. Non-functional BRCA1 leads to increased expression levels of S100A8 and S100A9 which then play a role in metastasis through binding to RAGE (Receptor for Advanced Glycation Endproducts) receptors on the surface of myeloid-derived suppressor cells [47–49]. While supporting literature as well as our data suggests a detectable increased expression in S100A8 and S100A9 in tear samples, the authors acknowledge a previous study on tears indicated reduced expression of S100A8 and S100A9 in pooled tear samples of breast cancer patients compared to normal patients [31]. However, this study does not provide a hypothesis for this contradictory expression profile and the variation in experimental parameters could be responsible for the observed differences (i.e. pooled samples versus individual sample evaluation, use of acetone protein precipitation methods, and evaluation of in-gel digestion versus in-solution trypsin digestion).

Galectin-3 binding protein (LG3BP) is a heavily glycosylated 90 kDa protein that is expressed in bodily secretions produced mostly by epithelial cells in glands, such as breast and tear ducts, as well as cancer cells [50]. LG3BP has been shown to be a binding site for proteins known to be involved in metastasis [51]. In addition, higher serum levels of LG3BP were associated with shorter survival in patients with breast carcinoma [52]. LG3BP was selected as a biomarker due to the elevated level of LG3BP observed by LC-MS/MS. However, ELISA data suggests a reduction in concentration in tears for breast cancer patients. A previous research group performed a comparison of vitamin-D binding protein concentrations in two different races using mass spectrometry, monoclonal and polyclonal ELISA kits [53]. They reported that these expression levels comparing the mass spectrometry results with polyclonal ELISA results had less than 9% variability but showed a higher (~85%) variability with monoclonal ELISA kits. They attributed this effect to the differential isoforms of the proteins detected using the two ELISA methods which varied by genotype. We believe that a similar difference in our ELISA and LC-MS/MS results could be attributed to the monoclonal ELISA kits used to quantify LG3BP.

The Area Under the Curve (AUC) for screening mammography has been reported to be anywhere from 0.67 to 0.84 depending on the modality used (digital vs. film), patient population, and breast density of participants [54,55]. We report an AUC of 0.902 with a sensitivity of 84.8%, a specificity of 86.4% and an accuracy of 85.6% and provides a strong starting point and justification for future research.

## Conclusions

The field of tear-based diagnostics is rapidly expanding beyond ocular diseases. Current studies have focused on detecting alterations in the tear proteome in a wide variety of systemic diseases ranging from Alzheimer’s to cancer [12,18,21,26,27,56]. Here, we provide an analysis of 273 individually collected tear samples. In this study, we examined the ocular proteome to identify protein biomarkers with altered expression levels in women diagnosed with breast cancer. Biomarker discovery was carried out using LC-MS/MS and selected markers were validated using ELISA. Our work provides data to support the growing body of evidence for continued evaluation of tear samples screening and diagnosis of systemic diseases. While the number of individual tear samples evaluated is large for the field of tear-based proteomics, it is quite small in the field of breast cancer as well as biomarker validation. Significantly larger studies would need to be conducted in order to reach a sound conclusion on the ability of tear proteins to distinguish between control and disease state samples.

Future investigations will focus on the additional biomarkers listed in Table 4 to aid in differentiating breast cancer from control; specifically, SPARC-like protein 1 and lactate dehydrogenase as these markers will provide more insight into ECM remodeling and metabolic processes respectively. Alternative approaches to both collection and sample processing procedures, such as exosome isolation, could allow for evaluation of not only intracellular and extracellular markers but also microRNA [57,58].
